# Antibacterial Efficacy of Local Therapeutic Agents Against Specific Gram-Positive Bacteria Associated with Pericoronitis

**DOI:** 10.3390/biomedicines14081700

**Published:** 2026-07-29

**Authors:** Özen Toranbeki, Mehmet Gagari Caymaz, Atalay Elver, Meryem Güvenir

**Affiliations:** 1 Department of Oral and Maxillofacial Surgery, Faculty of Dentistry, Kıbrıs Sağlık ve Toplum Bilimleri Üniversitesi (Cyprus Health and Social Sciences University), Mersin-10, Morphou 99700, Turkey; ozentoranbeki@gmail.com (Ö.T.); atalayelver1@gmail.com (A.E.); 2 Department of Medical Microbiology, Faculty of Medicine, Kıbrıs Sağlık ve Toplum Bilimleri Üniversitesi (Cyprus Health and Social Sciences University), Mersin-10, Morphou 99700, Turkey; meryemguvenir@hotmail.com

**Keywords:** pericoronitis, chlorhexidine, injectable platelet-rich fibrin, amoxicillin-loaded injectable platelet-rich fibrin, *Streptococcus mutans*, *Staphylococcus aureus*, *Enterococcus faecalis*

## Abstract

**Background/Objectives**: Pericoronitis is a polymicrobial, biofilm-associated odontogenic infection; however, selected facultative Gram-positive bacteria may contribute to oral infectious persistence. This study compared the residual antibacterial activity of chlorhexidine (CHX), low-level laser therapy (LLLT), injectable platelet-rich fibrin (i-PRF), and amoxicillin-loaded i-PRF against selected oral and odontogenic bacterial strains. **Methods**: Standardized planktonic suspensions of *Streptococcus mutans* ATCC 25175, *Staphylococcus aureus* ATCC 25923, and *Enterococcus faecalis* ATCC 29212 were prepared at 0.5 McFarland turbidity. Five conditions were evaluated: 0.2% CHX, i-PRF, amoxicillin-loaded i-PRF, 976 nm diode LLLT, and 0.9% sodium chloride as the negative control. Twelve independent tubes were used for each treatment and species, yielding 180 experimental units. After a residual 24-h exposure/incubation period, samples were serially diluted, plated on 5% sheep blood agar, and quantified as CFU/mL. Data were analyzed using non-parametric tests. **Results**: Significant treatment-dependent differences were observed for all species (*p* < 0.001). CHX showed the most consistent antibacterial activity, with median residual counts of 550 CFU/mL for *S. mutans*, 1 CFU/mL for *S. aureus,* and 5.5 CFU/mL for *E. faecalis.* i-PRF alone was comparable to the negative control. Amoxicillin-loaded i-PRF markedly reduced S. aureus counts, whereas *S. mutans* and *E. faecalis* remained at high residual levels. LLLT showed partial, variable inhibition mainly against *S. aureus*. **Conclusions**: CHX demonstrated the most consistent antibacterial effect in this planktonic residual 24-h in vitro model. i-PRF alone showed no measurable antibacterial activity, while amoxicillin-loaded i-PRF and LLLT exhibited limited, species-dependent effects.

## 1. Introduction

Dental infections are increasingly recognized as polymicrobial ecological disorders rather than diseases caused by a single pathogen. In oral biofilms, bacteria coexist within structured communities and interact through metabolic cooperation, local environmental modification, quorum sensing, and interspecies signaling, thereby increasing microbial persistence, pathogenicity, and antimicrobial tolerance [1,2]. The human oral and mucosal microbiome is niche-specific and dynamic, and disease may emerge when a balanced microbial community shifts toward dysbiosis [2,3].

Recent advances in oral microbiome research have shown that a substantial proportion of anaerobic and fastidious oral taxa remain insufficiently characterized because of their demanding cultivation requirements. As these previously underexplored organisms have become accessible to pure-culture analysis, distinctive structural and biological features have continued to emerge. A representative example is *Tannerella* sp. HOT-286, in which a prominent, potentially glycosylated extracellular S-layer was identified. This finding illustrates how newly characterized surface structures may contribute to niche-specific adaptation, ecological persistence, and the broader structural complexity of the oral microbiome, features that cannot be fully reproduced in simplified culture models [4]. This reinforces the need to interpret standardized in vitro models as partial representations of the broader oral infectious ecosystem.

Pericoronitis is a clinically relevant odontogenic infection that commonly develops around partially erupted mandibular third molars. Retention of plaque and food debris beneath the soft-tissue operculum creates a protected, low-oxygen microenvironment that favors the growth of facultative and obligate anaerobic microorganisms [5,6]. Although pericoronitis is typically considered a polymicrobial, anaerobic, and biofilm-associated condition, its microbial ecology cannot be reduced to a single bacterial group. Gram-negative anaerobes such as *Prevotella*, *Porphyromonas*, and *Fusobacterium* species are important components of odontogenic and periodontal infectious ecosystems, whereas facultative Gram-positive bacteria may participate in early colonization, ecological conditioning, and persistence within mixed oral communities [7,8].

The three organisms selected for the present study do not represent the full microbiological complexity of clinical pericoronitis. Instead, *Streptococcus mutans*, *Staphylococcus aureus*, and *Enterococcus faecalis* were used as standardized American Type Culture Collection (ATCC) reference strains to evaluate a selected facultative Gram-positive subcomponent of oral and odontogenic infections under controlled in vitro conditions [9,10,11,12].

*Streptococcus mutans* is a well-characterized oral biofilm-associated streptococcus that contributes to extracellular polysaccharide production, acidogenicity, and dental plaque ecology, while its surface-anchored and secreted proteins further promote adhesion, biofilm stability, and tolerance to environmental stress [3,9]. *Enterococcus faecalis* is clinically relevant because of its environmental tolerance, biofilm-forming ability, and persistence in oral and endodontic infections [10,11]. *Staphylococcus aureus* is not considered a primary etiological driver of pericoronitis; however, it is an opportunistic Gram-positive pathogen that may be relevant in oral surgical wounds, soft-tissue infections, and biomaterial-related antimicrobial testing [12]. Therefore, its inclusion in the present study was intended to broaden the assessment of facultative Gram-positive susceptibility rather than to imply that it is a dominant pericoronitis pathogen.

Chlorhexidine (CHX) remains one of the most widely used local antiseptic agents in dentistry because of its broad-spectrum antimicrobial activity and substantivity. Its cationic bisbiguanide structure promotes interaction with negatively charged bacterial surfaces, resulting in membrane disruption and the leakage of intracellular contents at effective concentrations [13,14]. Despite its strong antimicrobial profile, prolonged or repeated CHX use may be associated with tooth staining, taste alteration, mucosal irritation, and potential disturbance of oral microbial ecology [13,14,15]. Recent literature has discussed reduced chlorhexidine susceptibility and potential cross-resistance, although these issues were beyond the scope of the present study [16].

Low-level laser therapy (LLLT), also described within the broader framework of photobiomodulation, has been investigated in oral and maxillofacial applications because of its potential effects on inflammation, pain modulation, and tissue repair [17,18]. However, because laser–microorganism interactions are strongly influenced by dosimetry, irradiation geometry, medium characteristics, and bacterial species, the antibacterial outcomes of LLLT remain variable across experimental models [19,20].

Injectable platelet-rich fibrin (i-PRF) is an autologous platelet concentrate containing platelets, leukocytes, cytokines, growth factors, and a fibrin network. It is primarily used as a regenerative and immunomodulatory biomaterial, while its direct antimicrobial role remains less clearly established [21,22]. The antibacterial potential of platelet concentrates appears to be variable and may be influenced by preparation protocol, centrifugation parameters, cellular composition, leukocyte content, fibrin architecture, and the tested microorganism [23,24]. Accordingly, i-PRF should be interpreted as a biologically active scaffold with possible antimicrobial properties that require controlled validation, rather than as a predictable stand-alone disinfectant.

Combining platelet concentrates with antimicrobial agents has been proposed as an exploratory local carrier-based strategy that may integrate regenerative potential with antibacterial exposure. Previous in vitro studies have suggested that injectable platelet-rich fibrin can function as a drug carrier and may increase the antibacterial susceptibility of incorporated antibiotics under controlled conditions [25,26]. Amoxicillin remains a commonly used beta-lactam antibiotic in management. Incorporating amoxicillin into i-PRF may therefore provide a useful exploratory model for localized antibiotic loading; however, dose–response behavior, release kinetics, cytocompatibility, and clinical feasibility require further investigation before direct clinical recommendations can be made [25,26].

Based on this background, the present study was designed to compare the residual antibacterial activity of CHX, LLLT, i-PRF, and amoxicillin-loaded i-PRF with 0.9% sodium chloride as a negative control against selected facultative Gram-positive bacteria relevant to oral and odontogenic infections. The study was deliberately framed as a standardized planktonic in vitro residual 24-h exposure/incubation model rather than as a mature multispecies biofilm model or an acute clinical pericoronitis model.

The primary hypothesis was that at least one active treatment group would show a significant difference in residual CFU/mL values compared with the negative control after the 24-h exposure/incubation period. The secondary hypothesis was that no significant differences would be observed in residual CFU/mL values among the active treatment groups.

## 2. Materials and Methods

### 2.1. Study Design, Ethical Approval, and Experimental Setting

This study was designed as a prospective in vitro experimental study and was conducted at the Department of Oral and Maxillofacial Surgery, Cyprus Health and Social Sciences University, between 2024 and 2025. The study protocol was approved by the Cyprus Health and Social Sciences University Scientific Research Ethics Committee (Approval No. KSTU/2025/052). Written informed consent was obtained from all volunteers who donated blood for injectable platelet-rich fibrin (i-PRF) preparation. All procedures involving human-derived material were performed in accordance with the principles of the Declaration of Helsinki.

The experimental model was structured to evaluate the residual antibacterial effects of selected local therapeutic agents after a 24-h exposure/incubation period in a nutrient-supportive broth environment. Therefore, the present model should be interpreted as a residual 24-h exposure model rather than an acute 5-min contact-killing assay. This clarification is essential because chemical agents such as chlorhexidine and amoxicillin remained in the bacterial suspension during the post-treatment incubation period, whereas low-level laser therapy (LLLT) was applied as a single irradiation procedure without residual chemical activity.

The present study used standardized planktonic bacterial suspension models to enable controlled and reproducible comparison of the residual antibacterial activity of the tested local therapeutic agents. In this model, bacteria were evaluated as freely suspended cells in broth rather than as sessile microbial communities embedded within a mature biofilm matrix. Therefore, no mature mono-species or multispecies biofilm model was established. This design was selected as a preliminary in vitro screening approach to assess treatment-dependent bacterial responses under standardized experimental conditions. Accordingly, the findings should be interpreted as evidence of residual antibacterial activity against selected planktonic bacterial strains, rather than as a direct representation of the anaerobic, polymicrobial, biofilm-associated ecology of clinical pericoronitis.

### 2.2. Experimental Design, Group Allocation, and Replication

Five treatment groups were evaluated against three bacterial species. The treatment conditions were as follows: 0.2% chlorhexidine gluconate solution (CHX), injectable platelet-rich fibrin (i-PRF), i-PRF combined with amoxicillin, low-level laser therapy (LLLT), and 0.9% sodium chloride (NaCl) as the negative control.

For each bacterial species, 12 separately prepared and independently processed experimental tubes were used for each treatment group. Therefore, 60 experimental tubes were prepared for each bacterial species, resulting in a total of 180 experimental units across the entire study design (3 bacterial species × 5 treatment groups × 12 tubes).

The 12 replicates in each group did not represent repeated colony counts from a single tube. Instead, each replicate corresponded to a separate bacterial suspension tube that independently underwent the assigned treatment, incubation, serial dilution, plating, and colony-counting procedure. This design was used to reduce the risk of pseudoreplication and ensure that each colony-forming unit per milliliter (CFU/mL) value represented a distinct experimental unit.

A priori sample size calculation was performed using G*Power software (version 3.1.9.7). Because directly comparable preliminary data for the complete experimental design were limited, the calculation was based on a conservative medium effect size assumption (f = 0.29), with an alpha level of 0.05 and statistical power of 0.95. Under these assumptions, a total of 180 experimental units was considered sufficient for detecting treatment-related differences across the five treatment groups.

The experimental design, including bacterial species, group allocation, number of replicates, and incubation conditions, is summarized in Table 1.

### 2.3. Bacterial Strains and Culture Conditions

Standard reference bacterial strains were obtained from the American Type Culture Collection (ATCC) to ensure methodological standardization and reproducibility. The tested strains were *Streptococcus mutans* ATCC 25175, *Staphylococcus aureus* ATCC 25923, and *Enterococcus faecalis* ATCC 29212. These strains were selected to represent a standardized facultative Gram-positive subcomponent of oral and odontogenic infections, and their microbiological characteristics, clinical relevance, and inclusion rationale are presented in Table 2.

Bacterial suspensions were prepared in Brain Heart Infusion (BHI) broth (Sigma-Aldrich, St. Louis, MO, USA) under aseptic conditions. The turbidity of each suspension was adjusted to the 0.5 McFarland standard, corresponding to approximately 1.5 × 10^8^ CFU/mL, using a nephelometer (SN 070500012093, Becton, Dickinson and Company, Sparks, MD, USA). Following standardization, bacterial suspensions were incubated at 37 °C for 24 h to obtain actively growing planktonic cultures before treatment application. Cultures showing abnormal turbidity, sedimentation, discoloration, or any sign of contamination were excluded and freshly prepared. The antibacterial response of each strain was assessed using a standardized planktonic suspension model, in which residual bacterial viability after treatment and 24-h incubation was quantified as CFU/mL under controlled in vitro conditions.

### 2.4. Randomization and Blinding

Experimental tubes were assigned to treatment groups using a computer-generated randomization sequence. Each tube was labeled with a unique code before treatment application. Colony counting was performed by an investigator blinded to group allocation in order to reduce observer bias.

### 2.5. General Treatment Procedure

To standardize the initial bacterial load, all treatment groups were prepared using 5 mL of standardized planktonic bacterial suspension. In the CHX, NaCl, i-PRF, and i-PRF + amoxicillin groups, 1 mL of the relevant solution or preparation was added to the bacterial suspension. In the LLLT group, no additional liquid agent was added; therefore, the samples were irradiated directly as 5 mL standardized bacterial suspensions.

After treatment application, all samples were incubated at 37 °C for 24 h before serial dilution and plating. No chemical neutralization step was performed after treatment application. Accordingly, the chemical treatment groups were evaluated as residual 24-h exposure conditions, whereas the LLLT group was evaluated as a post-irradiation residual viability condition. Because bacterial viability was expressed as CFU/mL, the results were reported in a volume-normalized manner.

### 2.6. Chlorhexidine Group

In the CHX group, 1 mL of commercially available 0.2% chlorhexidine gluconate solution (Klorhex^®^, Drogsan, Turkey) was added to 5 mL of the standardized bacterial suspension, resulting in a total assay volume of 6 mL. The mixture was gently homogenized and incubated at 37 °C for 24 h before microbiological analysis. CHX was included because of its well-established broad-spectrum antibacterial activity and frequent use as a local antiseptic agent in dental practice. The reported CHX concentration refers to the concentration of the commercial solution added to the bacterial suspension.

### 2.7. Injectable Platelet-Rich Fibrin Group

Injectable platelet-rich fibrin was freshly prepared from healthy volunteers immediately before each experimental session. All donors were systemically healthy ASA I individuals aged 18–28 years. Venous blood was collected in sterile 10 mL glass tubes without an anticoagulant and was centrifuged immediately after collection at 700 rpm, approximately 60× *g*, for 3 min using a Neuation iFuge L30P centrifuge (Neuation Technologies, Pvt. Ltd., Gandhinagar, Gujarat, India).

After centrifugation, the upper liquid i-PRF fraction was carefully collected under aseptic conditions. For the i-PRF group, 1 mL of freshly prepared i-PRF was added to 5 mL of standardized planktonic bacterial suspension. The mixture was gently homogenized and incubated at 37 °C for 24 h before microbiological analysis.

All i-PRF samples were used within 10 min of preparation. No i-PRF sample was stored, frozen, or reused. This approach was chosen to preserve the biological characteristics of the freshly obtained autologous platelet concentrate and minimize variability related to storage or delayed application.

### 2.8. i-PRF + Amoxicillin Group

In the i-PRF + amoxicillin group, freshly prepared i-PRF was used as an autologous carrier for amoxicillin. Amoxicillin was selected because it is a commonly used first-line beta-lactam antibiotic in the management of odontogenic infections when antibiotic therapy is indicated. Amoxicillin was incorporated into 1 mL of i-PRF using commercially available Largopen^®^ oral suspension (Bilim İlaç San. ve Tic. A. Ş., Kapaklı, Tekirdağ, Turkey) to obtain a nominal loading concentration of 10 µg/mL relative to the i-PRF carrier volume. The preparation was gently vortexed to obtain a homogeneous amoxicillin-loaded i-PRF mixture.

Subsequently, a 1 mL aliquot of the amoxicillin-loaded i-PRF preparation was added to 5 mL of standardized bacterial suspension, resulting in a total assay volume of 6 mL. The mixture was gently homogenized and incubated at 37 °C for 24 h before serial dilution and plating. This group was included to evaluate an exploratory i-PRF-based local antibiotic-loading approach under the residual 24-h exposure model.

### 2.9. Low-Level Laser Therapy Group

In the LLLT group, no additional liquid agent was added to the bacterial suspension. The standardized 5 mL planktonic bacterial suspension was irradiated directly using a 976 nm diode laser device (LX16 Plus, Guilin Woodpecker Medical Instrument Co., Ltd., Guilin, Guangxi, China).

The laser parameters were as follows: wavelength 976 nm, continuous-wave mode, output power 1 W, irradiation time 60 s, total delivered energy 60 J, and energy density approximately 60 J/cm^2^. The irradiation area was standardized to approximately 1 cm^2^, and the laser tip was positioned perpendicular to the tube opening at a fixed distance of 1 cm. The laser output was checked before the experimental procedures to ensure consistent irradiation conditions.

After irradiation, the samples were incubated at 37 °C for 24 h before microbiological analysis. As no residual chemical antimicrobial agent was added to the LLLT group, this condition was evaluated as a post-irradiation residual viability model under the same 24-h incubation framework used for the other experimental groups.

### 2.10. Negative Control Group

In the negative control group, 1 mL of sterile 0.9% sodium chloride solution was added to 5 mL of standardized bacterial suspension. The mixture was gently homogenized and incubated at 37 °C for 24 h under the same conditions as the chemical treatment groups. This group was used to evaluate bacterial growth in the absence of an active antimicrobial agent.

### 2.11. Serial Dilution, Plating, and Colony Counting

After the 24-h exposure/incubation period, each sample was serially diluted tenfold in sterile saline from 10^−1^ to 10^−6^. From each dilution, 10 µL was plated onto 5% sheep blood agar plates (Sigma-Aldrich, St. Louis, MO, USA) under aseptic conditions. The plates were incubated at 37 °C for 24 h.

**Figure 1:**
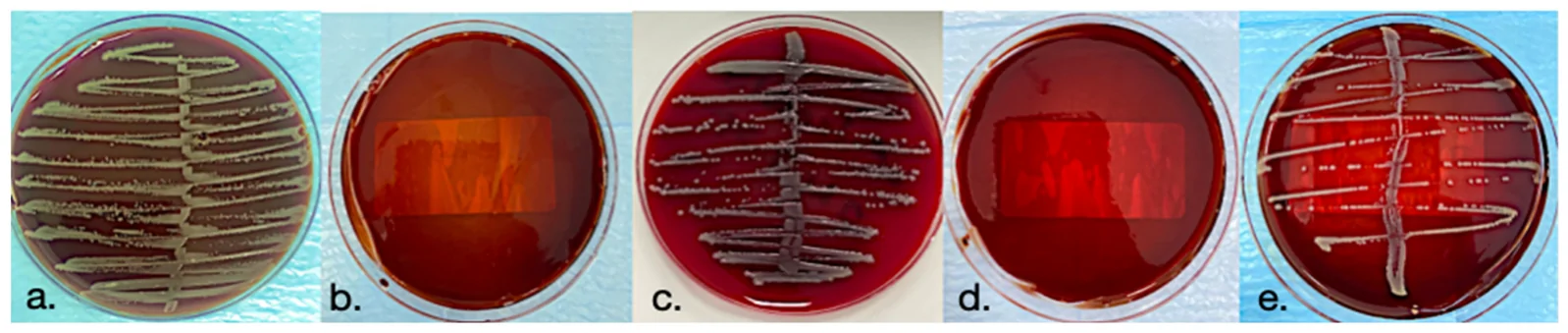
Representative post-incubation bacterial colony growth observed on 5% sheep blood agar plates in the (**a**) NaCl, (**b**) CHX, (**c**) i-PRF, (**d**) i-PRF + amoxicillin, and (**e**) LLLT groups, respectively.

After incubation, visible colonies were manually counted under blinded conditions and expressed as CFU/mL (Figure 1). Plates showing confluent growth or colonies that were too numerous to count were recorded using a predefined upper reporting value of 1.0 × 10^8^ CFU/mL.

### 2.12. Statistical Analysis

Statistical analyses were performed using IBM SPSS Statistics (Version 28.0, IBM Corp., Armonk, NY, USA). Since the distribution of colony-forming unit (CFU/mL) values did not satisfy the assumptions of normality, non-parametric methods were applied throughout the analyses. Descriptive statistics are presented as median, interquartile range (IQR), and minimum–maximum values. To account for the upper quantification limit of the assay, the number of samples reaching the predefined ceiling value (1 × 10^8^ CFU/mL) was additionally reported for each treatment group.

For each bacterial species, residual CFU/mL values were compared among treatment groups using the Kruskal–Wallis test. When a significant overall difference was detected, pairwise comparisons were performed using Bonferroni-adjusted Mann–Whitney U tests. In addition, within each treatment group, bacterial species were compared using the Kruskal–Wallis test followed by Bonferroni-adjusted pairwise Mann–Whitney U tests where appropriate. Effect sizes were calculated using epsilon-squared (ε^2^) to quantify the magnitude of group differences. Statistical significance was defined as *p* < 0.05.

**Figure 2:**
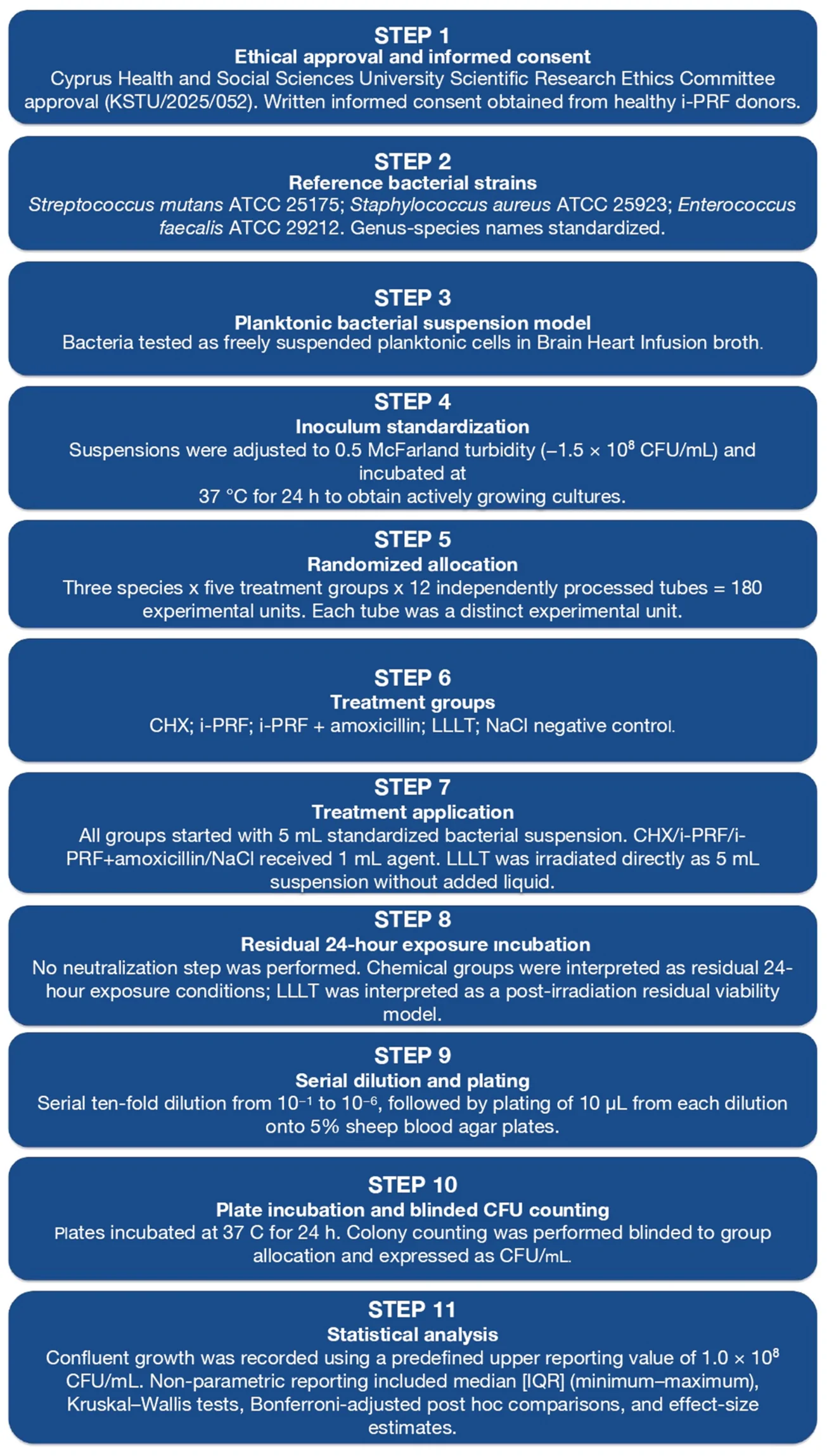
Methodological flowchart.

An overview of the complete methodological workflow, from experimental allocation to CFU/mL quantification and statistical evaluation, is provided in Figure 2.

## 3. Results

### 3.1. Overview of Treatment-Dependent Antibacterial Effects

The residual antibacterial effects of the tested local therapeutic agents were evaluated after the 24-h exposure/incubation period using colony-forming unit per milliliter (CFU/mL) values. Because the data were not normally distributed and several high-growth groups reached the predefined upper recording limit, the results were primarily summarized using median, interquartile range (IQR), and minimum–maximum values. Significant treatment-dependent differences were observed within all three bacterial species. The Kruskal–Wallis test showed significant differences among treatment groups for *Streptococcus mutans* (H = 58.07, df = 4, *p* < 0.001, ε^2^ = 0.983), *Staphylococcus aureus* (H = 41.80, df = 4, *p* < 0.001, ε^2^ = 0.687), and *Enterococcus faecalis* (H = 50.82, df = 4, *p* < 0.001, ε^2^ = 0.851). These findings indicate that the residual antibacterial effects of the tested applications differed substantially among treatment groups for each bacterial species.

### 3.2. Treatment Effects on Streptococcus mutans

For *S. mutans,* chlorhexidine (CHX) produced the greatest reduction in residual bacterial counts. The CHX group showed a median CFU/mL value of 550 [IQR: 7.75–257,500] (range: 1–1 × 10^6^), whereas LLLT, NaCl, i-PRF, and i-PRF + amoxicillin all remained at the predefined upper recording limit, with median values of 1 × 10^8^ CFU/mL.

Bonferroni-adjusted post hoc comparisons showed that CHX resulted in significantly lower CFU/mL values than all other treatment groups. In contrast, no significant differences were observed among LLLT, NaCl, i-PRF, and i-PRF + amoxicillin. These findings indicate that under the present residual 24-h planktonic model, CHX was the only treatment associated with a significant reduction in residual *S. mutans* counts.

### 3.3. Treatment Effects on Staphylococcus aureus

For *S. aureus,* a more treatment-specific response pattern was observed. CHX showed the lowest residual bacterial counts, with a median value of 1 [IQR: 1–1] CFU/mL (range: 1–1 × 10^8^). The i-PRF + amoxicillin group also showed low residual counts, with a median value of 10 [IQR: 1–100] CFU/mL (range: 1–1 × 10^8^). LLLT showed an intermediate and more variable response, with a median value of 55,000 [IQR: 775–1 × 10^8^] CFU/mL (range: 10–1 × 10^8^).

Post hoc analyses indicated that CHX produced significantly lower CFU/mL values than LLLT, NaCl, and i-PRF. However, the difference between CHX and i-PRF + amoxicillin did not reach statistical significance after Bonferroni correction. The i-PRF + amoxicillin group showed significantly lower bacterial counts than LLLT, NaCl, and i-PRF, indicating a species-dependent response mainly against *S. aureus*. LLLT also differed significantly from NaCl, supporting a partial inhibitory effect, although its response was less consistent than that of CHX and i-PRF + amoxicillin.

### 3.4. Treatment Effects on Enterococcus faecalis

For *E. faecalis,* CHX demonstrated the strongest antibacterial effect. The median residual count in the CHX group was 5.5 [IQR: 1–10] CFU/mL (range: 1–100). In contrast, NaCl, i-PRF, and i-PRF + amoxicillin remained at the predefined upper reporting value, with median values of 1 × 10^8^ CFU/mL. The LLLT group also showed high residual bacterial counts, with a median value of 1 × 10^8^ [IQR: 7.50 × 10^7^–1 × 10^8^] CFU/mL (range: 100–1 × 10^8^).

Bonferroni-adjusted post hoc comparisons showed that CHX resulted in significantly lower CFU/mL values than all other treatment groups. No significant differences were detected among LLLT, NaCl, i-PRF, and i-PRF + amoxicillin. These results indicate that CHX was the only treatment associated with a significant reduction in residual *E. faecalis* counts under the present experimental conditions.

Residual CFU/mL values for all bacterial species and treatment groups after treatment exposure and 24-h incubation are summarized in Table 3.

### 3.5. Inter-Species Comparisons Within Each Treatment Group

To further evaluate species-dependent differences in bacterial survival, inter-species comparisons of residual CFU/mL values were performed within each treatment group after the 24-h residual exposure/incubation period, as shown in Table 4.

In the CHX group, a statistically significant inter-species difference was observed (H = 9.21, df = 2, *p* = 0.010, ε^2^ = 0.218). Pairwise comparisons indicated that this difference was mainly associated with lower residual counts in *S. aureus* compared with *S. mutans*.

In the LLLT group, inter-species differences were also significant (H = 10.09, df = 2, *p* = 0.006, ε^2^ = 0.245). This difference was primarily related to the lower median CFU/mL value observed for *S. aureus* compared with *S. mutans,* whereas *E. faecalis* remained closer to the high-growth range.

No significant inter-species difference was observed in the NaCl group (H = 0.00, df = 2, *p* = 1.000, ε^2^ = 0.000), indicating similar bacterial growth across the tested species in the absence of an active antimicrobial agent. Similarly, i-PRF alone did not produce a statistically significant inter-species difference (H = 4.11, df = 2, *p* = 0.128, ε^2^ = 0.064).

In contrast, the i-PRF + amoxicillin group showed a significant inter-species difference (H = 29.67, df = 2, *p* < 0.001, ε^2^ = 0.838). This difference was driven by the lower residual counts observed for *S. aureus,* whereas *S. mutans* and *E. faecalis* remained at the predefined upper reporting value. These findings indicate that the antibacterial response to amoxicillin-loaded i-PRF was species-dependent and was most evident against *S. aureus*.

### 3.6. Ceiling Effect and Interpretation of High-Growth Groups

**Figure 3:**
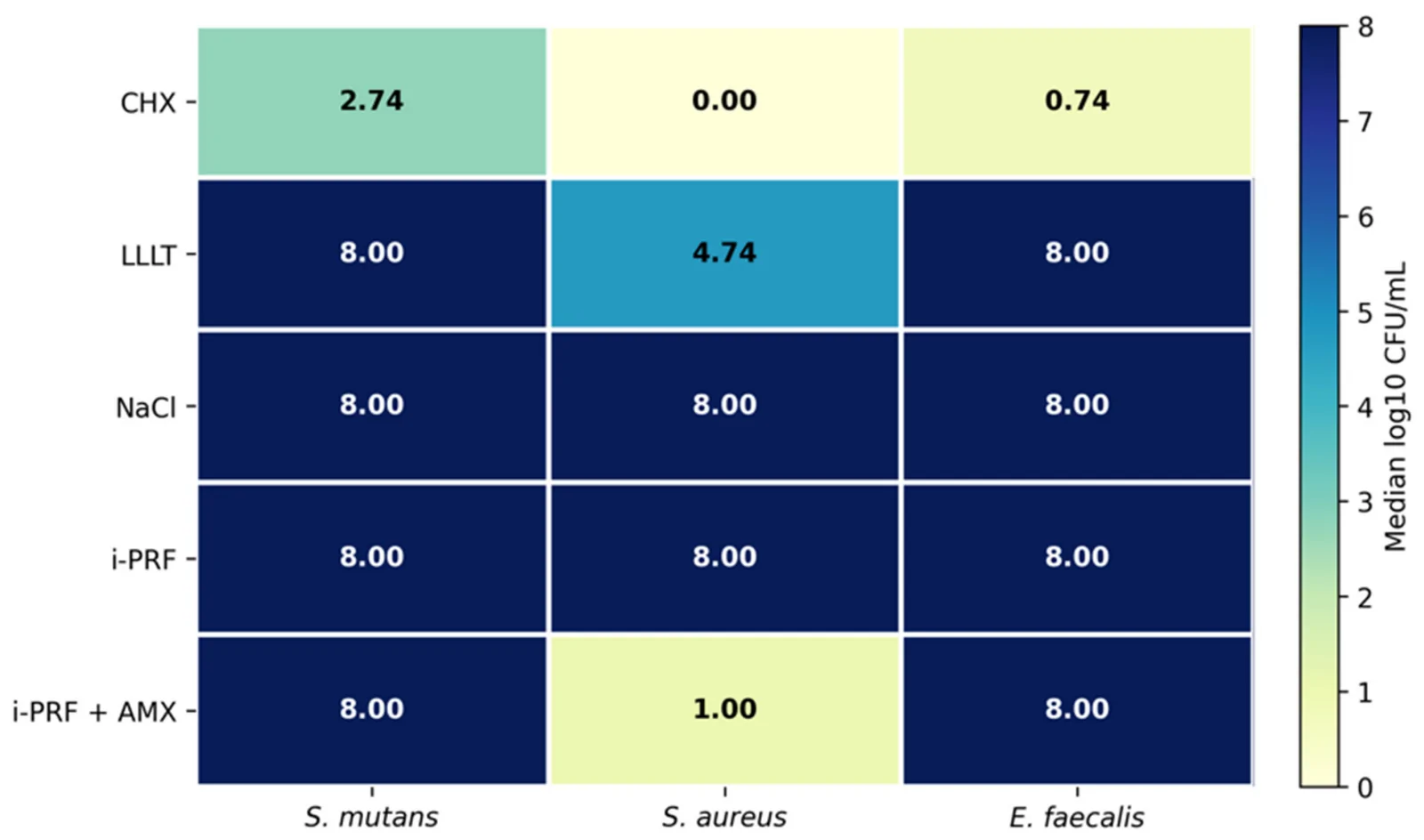
Heatmap of median log10-transformed residual CFU/mL values after treatment exposure and 24-h incubation.

Several high-growth groups reached the predefined upper recording limit of 1 × 10^8^ CFU/mL. This ceiling effect was particularly evident in the NaCl and i-PRF groups, where most or all replicates remained at the upper limit across the tested species. Similar ceiling-level growth was also observed for *S. mutans* and *E. faecalis* in the i-PRF + amoxicillin group, as well as for *S. mutans* and *E. faecalis* in the LLLT group. Importantly, some groups with low median values also included isolated ceiling-level observations. For example, the CHX and i-PRF + amoxicillin groups against *S. aureus* each included one replicate recorded at 1 × 10^8^ CFU/mL, which substantially inflated the arithmetic mean and standard deviation despite very low median values. Therefore, median and IQR values were considered more appropriate than mean ± standard deviation for interpreting the antibacterial response. Overall, CHX demonstrated the most consistent antibacterial activity across all tested species. The i-PRF + amoxicillin group showed a pronounced species-dependent effect, mainly against *S. aureus.* LLLT produced a partial and variable inhibitory effect against *S. aureus,* whereas i-PRF alone showed no measurable antibacterial activity compared with the negative control. The overall distribution of median log10-transformed residual CFU/mL values across the bacterial species and treatment groups is presented in Figure 3.

The heatmap illustrates the median log10-transformed CFU/mL values for each treatment group and bacterial species. Lower values indicate lower residual bacterial viability, whereas values approaching 8 indicate groups reaching the predefined upper recording limit of 1 × 10^8^ CFU/mL. A value of 0.00 corresponds to a median count of 1 CFU/mL after log10 transformation and should not be interpreted as complete absence of bacterial growth. CHX showed the most consistent reduction across all tested species. i-PRF + amoxicillin demonstrated a species-dependent reduction mainly against *Staphylococcus aureus,* whereas i-PRF alone and NaCl remained close to the upper recording limit across all species.

Several high-growth groups reached the predefined upper recording limit of 1 × 10^8^ CFU/mL. This ceiling effect was particularly evident in the NaCl and i-PRF groups, where most or all replicates remained at the upper limit across the tested species. Similar ceiling-level growth was also observed for *Streptococcus mutans* and *Enterococcus faecalis* in the i-PRF + amoxicillin group, as well as for *S. mutans* and *E. faecalis* in the LLLT group. Importantly, some groups with low median values also included isolated ceiling-level observations. For example, the CHX and i-PRF + amoxicillin groups against *S. aureus* each included one replicate recorded at 1 × 10^8^ CFU/mL, which substantially inflated the arithmetic mean and standard deviation despite very low median values. Therefore, median and IQR values were considered more appropriate than mean ± standard deviation for interpreting the antibacterial response. Overall, CHX demonstrated the most consistent antibacterial activity across all tested species. The i-PRF + amoxicillin group showed a pronounced species-dependent effect, mainly against *S. aureus*. LLLT produced a partial and variable inhibitory effect against *S. aureus,* whereas i-PRF alone showed no measurable antibacterial activity compared with the negative control.

## 4. Discussion

The present study was designed to evaluate whether different local therapeutic modalities exerted measurable antibacterial effects against selected Gram-positive facultative bacteria under a planktonic 24-h residual exposure/incubation model. Significant treatment-dependent differences in residual colony-forming units per milliliter (CFU/mL) values were observed for all tested bacterial species, indicating that the antibacterial outcomes were influenced by the applied modality. Therefore, the primary hypothesis was accepted, as at least one active treatment group demonstrated a significant difference in residual CFU/mL values compared with the negative control. In contrast, the secondary hypothesis was not accepted, because the active treatment groups did not exhibit equivalent antibacterial efficacy. Among the tested interventions, chlorhexidine (CHX) showed the most consistent and pronounced antibacterial activity across *Streptococcus mutans*, *Staphylococcus aureus*, and *Enterococcus faecalis*, whereas injectable platelet-rich fibrin (i-PRF) alone yielded residual bacterial counts comparable to those of the negative control. Amoxicillin-loaded i-PRF and low-level laser therapy (LLLT) showed limited and species-dependent effects, particularly against *S. aureus.* These findings suggest that within the limitations of this in vitro model, the antibacterial performance of the tested local agents differed substantially according to both treatment modality and bacterial species.

These findings should be interpreted in light of the experimental scope of the present study. The in vitro model was designed to evaluate the antibacterial response of selected Gram-positive facultative species under standardized planktonic conditions. Therefore, it should not be regarded as a complete microbiological simulation of clinical pericoronitis, but rather as a controlled preliminary model focusing on a limited bacterial subset relevant to oral and odontogenic infections.

This distinction is important because pericoronitis is a polymicrobial, biofilm-associated odontogenic infection in which anaerobic Gram-negative organisms, facultative bacteria, host-related factors, local tissue anatomy, and subopercular environmental conditions interact dynamically [1,2,5,6,7,8]. Accordingly, the selected organisms should be understood as a standardized facultative Gram-positive subcomponent of a broader oral infectious ecology, rather than as the complete microbial representation of pericoronitis. Moreover, oral biofilms differ substantially from planktonic cultures in terms of microbial organization, extracellular matrix protection, metabolic heterogeneity, oxygen gradients, and antimicrobial tolerance [1,2,20].

The pronounced antibacterial effect observed in the CHX group is consistent with the established broad-spectrum activity of chlorhexidine, which acts by interacting with bacterial cell surfaces and disrupting membrane integrity [13,14]. In the present 24-h residual exposure/incubation model, CHX produced the lowest median residual CFU/mL values across all tested species, including S. mutans, S. aureus, and *E. faecalis*. These findings indicate that CHX provided the most consistent antibacterial activity against the selected planktonic Gram-positive organisms under the experimental conditions of this study. However, antimicrobial efficacy alone is insufficient to determine clinical suitability, particularly because cytocompatibility was not assessed in the present study. Therefore, the clinical interpretation of CHX-related findings should also consider its known local adverse effects and potential influence on the oral microbial balance [13,14,15].

In contrast to CHX, i-PRF alone showed no measurable antibacterial activity in the present model. Median CFU/mL values remained close to the upper recording limit across all tested species, similar to the negative control. These findings suggest that under the tested conditions, the specific i-PRF protocol used in this study did not produce a detectable antibacterial response and did not act as a reliable stand-alone antimicrobial agent against the selected Gram-positive organisms.

This finding should be interpreted in relation to the primary biological role of i-PRF. Injectable platelet-rich fibrin is an autologous platelet concentrate containing platelets, leukocytes, cytokines, growth factors, and a fibrin matrix, and its main clinical relevance is generally associated with tissue repair, angiogenesis, immune modulation, and wound healing rather than direct antimicrobial action [21,22]. Although antibacterial effects of PRF-based preparations have been reported, recent studies have shown that this activity may vary according to preparation protocol, centrifugation parameters, leukocyte and platelet content, fibrin architecture, donor-related biological factors, and bacterial species [23,24]. In particular, Çetin et al. reported antibacterial effects of leukocyte- and platelet-rich fibrin against *Escherichia coli* and *Enterococcus faecalis*, whereas Popowski et al. demonstrated variable in vitro antibacterial activity of platelet-rich fibrin obtained from healthy individuals against selected oral pathogenic bacteria [27,28]. Therefore, the absence of measurable antibacterial activity in the i-PRF-alone group in the present study should not be interpreted as evidence of a universal lack of antimicrobial potential, but rather as a finding related to the specific preparation protocol and planktonic experimental conditions used in this model.

The amoxicillin-loaded i-PRF group showed a species-dependent antibacterial response. This group produced a marked reduction in *S. aureus* counts, with a median residual value of 10 CFU/mL, whereas *S. mutans* and *E. faecalis* remained close to the upper recording limit. Notably, the response against S. aureus was statistically comparable to that of CHX under the adjusted post hoc comparison, suggesting a measurable carrier-associated antibacterial effect for this species. However, this comparable response was not observed across all tested organisms. Therefore, the effect of amoxicillin-loaded i-PRF should be interpreted as species-dependent rather than uniformly equivalent to CHX.

Beyond the species-specific antibacterial response observed in the present study, the amoxicillin-loaded i-PRF approach may also be interpreted within the broader framework of biomaterial-based localized therapeutic delivery, which is increasingly being explored to enhance site-specific drug exposure while potentially limiting systemic drug burden and unintended effects on host-associated microbial communities [29,30]. The response observed in the amoxicillin-loaded i-PRF group may be considered within the broader concept of localized biological delivery. Platelet-derived fibrin matrices and antibiotic-loaded PRF preparations have been proposed as potential local carriers for antimicrobial agents [25,26]. In this context, Bilginaylar et al. reported that platelet-rich fibrin-mediated local antibiotic delivery may improve outcomes in impacted mandibular third molar surgery, while Niemczyk et al. systematically summarized in vitro evidence supporting antibiotic-loaded PRF as a potential antimicrobial carrier system [31,32]. Accordingly, the selective reduction observed with amoxicillin-loaded i-PRF may support further the investigation of i-PRF as an autologous fibrin-based carrier for local antibiotic delivery [29]. Nevertheless, this interpretation remains exploratory, because the present study did not evaluate the amoxicillin release kinetics, diffusion behavior, degradation profile, or sustained-release capacity from the i-PRF matrix. Therefore, no conclusion can be drawn regarding controlled or prolonged amoxicillin release. Future studies assessing the release kinetics, dose–response behavior, antimicrobial stability, and biofilm-based models are required before i-PRF can be defined as a controlled local drug delivery system.

However, because the amoxicillin release kinetics, clinically validated local concentrations, acute contact efficacy, and cytocompatibility were not assessed, this approach should be interpreted as an exploratory strategy requiring further dose–response, biofilm-based, and cytocompatibility studies.

LLLT produced a partial and species-dependent antibacterial response in the present study. A reduction in residual CFU/mL was observed mainly against *S. aureus*, whereas *S. mutans* and *E. faecalis* remained close to the upper recording limit after 24 h. This pattern is consistent with the parameter-dependent nature of laser–microorganism interactions. The antibacterial effect of diode laser irradiation may vary according to wavelength, power output, irradiation time, energy density, beam geometry, distance from the target, optical penetration, characteristics of the surrounding medium, and bacterial species [18,33]. In the present experiment, a 976 nm diode laser was applied at 1 W for 60 s over an approximately 1 cm^2^ irradiation area. Unlike chemical antimicrobial agents, LLLT does not provide a residual active compound after irradiation. Therefore, bacteria that survived the initial laser exposure may have continued to proliferate during the subsequent 24-h incubation period. Accordingly, the LLLT findings should be interpreted as post-irradiation residual viability results rather than as evidence of continuous antimicrobial exposure.

The absence of a sustained reduction in *E. faecalis* and *S. mutans* after LLLT in the present study should be interpreted in relation to the specific irradiation protocol used. In this model, the 976 nm diode laser was applied externally at 1 W for 60 s over an approximately 1 cm^2^ area, followed by 24-h incubation in broth. Under these conditions, residual bacterial growth was not consistently suppressed for *E. faecalis* or *S. mutans*. However, previous in vitro and ex vivo studies have reported antibacterial effects against these organisms using different experimental configurations, including altered power settings, longer or repeated irradiation periods, different irradiation modes, closer or intraluminal fiber delivery, and higher effective energy transfer to the bacterial target [19,33,34]. These methodological differences may explain why reductions were observed in some previous studies but were not consistently reproduced in the present planktonic broth model.

From a statistical perspective, interpretation of the CFU/mL data required careful consideration because several groups included ceiling-level observations at the predefined upper recording limit of 1 × 10^8^ CFU/mL. In some groups, particularly the CHX and i-PRF + amoxicillin groups against *S. aureus*, isolated ceiling-level values increased the arithmetic mean and standard deviation despite very low median counts. Therefore, median, interquartile range, and minimum–maximum values were considered more appropriate than mean ± standard deviation for describing the non-normally distributed data. The inclusion of ceiling n/12 and effect size estimates further supported a more transparent interpretation of the treatment response beyond *p*-values. However, because confluent growth was recorded at the predefined upper limit of 1 × 10^8^ CFU/mL, small differences among high-growth groups may not have been fully captured. Therefore, groups such as NaCl, i-PRF, and some LLLT or i-PRF + amoxicillin conditions should be interpreted as showing no measurable reduction under the present assay conditions, rather than as being biologically identical. Future studies using extended dilution ranges, automated colony counting, optical density monitoring, viability assays, or molecular quantification may provide greater resolution among high-growth groups.

In terms of clinical applicability, the present findings should be interpreted as preliminary in vitro comparative data rather than as a basis for a clinical treatment protocol for pericoronitis. The model did not reproduce key clinical factors such as saliva, tissue interaction, host response, mechanical debridement, anaerobic niches, biofilm architecture, or obligate anaerobic species commonly associated with pericoronitis, including *Prevotella*, *Fusobacterium*, and *Porphyromonas* [5,6,7,8].

Overall, within the limitations of the present in vitro model, CHX showed the most consistent antibacterial activity against the tested planktonic Gram-positive species. i-PRF alone did not produce a measurable antibacterial effect, while amoxicillin-loaded i-PRF exhibited selective activity mainly against *S. aureus*, and LLLT produced a partial, parameter-dependent response. Thus, the present study provides a standardized in vitro comparison of selected local therapeutic approaches and fulfills its primary aim by characterizing treatment- and species-dependent antibacterial response patterns against selected planktonic Gram-positive bacteria relevant to the broader pericoronitis-associated microbial ecology.

### Limitations

The present study has several limitations that should be acknowledged. First, the experimental model was based on planktonic bacterial suspensions rather than mature mono-species or multispecies biofilms. Since biofilm-embedded bacteria generally exhibit higher antimicrobial tolerance than planktonic cells, the findings should be interpreted as preliminary in vitro screening data rather than direct evidence of clinical biofilm control [20]. Second, only three standardized facultative Gram-positive ATCC strains, namely *Streptococcus mutans* ATCC 25175, *Staphylococcus aureus* ATCC 25923, and *Enterococcus faecalis* ATCC 29212, were tested. Although these species are relevant to oral and odontogenic infections, they do not represent the full polymicrobial ecology of pericoronitis, particularly the contribution of obligate anaerobes, Gram-negative species, spirochetes, and interspecies interactions [5,6,7,8]. Third, the residual 24-h exposure/incubation design does not fully reproduce clinical exposure conditions. Because no neutralization step was performed after treatment application, the chemical groups reflect prolonged residual exposure rather than short-term contact-killing activity.

Additional limitations are related to the specific treatment protocols. The i-PRF findings are limited to a single preparation protocol, and leukocyte concentration, platelet concentration, fibrin architecture, growth factor levels, and antimicrobial peptide release were not assessed. Similarly, the amoxicillin-loaded i-PRF group was evaluated as an exploratory carrier-based condition without an analysis of the release kinetics, dose–response behavior, local concentration stability, or cytocompatibility. The LLLT results are also limited to a single diode laser protocol, although antibacterial outcomes may vary according to wavelength, power, energy density, irradiation time, beam geometry, delivery method, medium characteristics, and target organism [33,35]. Furthermore, antibacterial activity was assessed without evaluating cytocompatibility and photothermal effects on host cells relevant to oral wound healing and regeneration. Finally, recording confluent bacterial growth as 1 × 10^8^ CFU/mL introduced a ceiling effect that may have limited discrimination among high-growth groups. Future studies should incorporate biofilm-based and polymicrobial models, anaerobic species, extended dilution ranges, complementary viability assays, cytocompatibility testing, and protocol-specific optimization to better define the translational relevance of these local therapeutic approaches.

## 5. Conclusions

Within the limitations of this planktonic residual 24-h in vitro model, CHX demonstrated the most consistent antibacterial activity against the tested facultative Gram-positive bacteria. i-PRF alone showed no measurable antibacterial activity under the preparation protocol and experimental conditions used in this study. Amoxicillin-loaded i-PRF and LLLT showed limited, species-dependent antibacterial effects mainly against *Staphylococcus aureus*, while *Streptococcus mutans* and *Enterococcus faecalis* were not consistently reduced by these approaches.

These findings support the use of CHX as an effective antimicrobial comparator in controlled in vitro screening, but they do not establish clinical superiority or routine clinical applicability. The results also suggest that i-PRF-based antibiotic loading and laser-based approaches may warrant further investigation as adjunctive experimental strategies; however, future studies should include anaerobic multispecies biofilm models, dose–response analysis, release kinetics, cytocompatibility testing, and clinically relevant exposure conditions before translation to pericoronitis management can be considered.

## Figures and Tables

**Table 1:** Overview of the experimental design, sample allocation, and incubation conditions.

Bacterial Species	Treatment Groups	Replicates per Group	Total Tubes per Species	Incubation Temperature (°C)	Incubation Time (h)
*Streptococcus mutans*	5	12	60	37	24
*Staphylococcus aureus*	5	12	60	37	24
*Enterococcus faecalis*	5	12	60	37	24
Total	—	—	180	—	—

**Table 2:** Bacterial strains used in the present study and rationale for inclusion.

Bacterial Species	ATCC Strain	Gram Reaction/Oxygen Requirement	Clinical and Microbiological Relevance	Rationale for Inclusion
*Streptococcus mutans*	ATCC 25175	Gram-positive; facultative anaerobe	Oral biofilm-associated streptococcus involved in dental plaque ecology, extracellular polysaccharide production and acidogenicity.	Selected as a standardized oral streptococcal model representing part of the facultative Gram-positive component of oral biofilm-associated infections.
*Staphylococcus aureus*	ATCC 25923	Gram-positive; facultative anaerobe	Opportunistic pathogen relevant to oral surgical wounds, soft-tissue infection risk and antimicrobial testing.	Included as a clinically relevant opportunistic Gram-positive species; not presented as a dominant etiological driver of pericoronitis.
*Enterococcus faecalis*	ATCC 29212	Gram-positive; facultative anaerobe	Persistent oral/endodontic organism characterized by environmental tolerance and biofilm-forming capacity.	Selected as a resistant facultative Gram-positive organism relevant to persistent odontogenic and oral infections.

**Table 3:** Treatment-dependent residual CFU/mL values for each bacterial species after treatment exposure and 24-h incubation.

Treatment	*Streptococcus mutans* Median [IQR] (Min–Max); Ceiling n/12	*Staphylococcus aureus* Median [IQR] (Min–Max); Ceiling n/12	*Enterococcus faecalis* Median [IQR] (Min–Max); Ceiling n/12
**CHX**	550 [7.75–257,500] (1–1 × 10^6^); 0/12 ^a^	1 [1–1] (1–1 × 10^8^); 1/12 ^a^	5.5 [1–10] (1–100); 0/12 ^a^
**LLLT**	1 × 10^8^ [1 × 10^8^–1 × 10^8^] (1 × 10^8^–1 × 10^8^); 12/12 ^b^	55,000 [775–1 × 10^8^] (10–1 × 10^8^); 5/12 ^b^	1 × 10^8^ [7.50 × 10^7^–1 × 10^8^] (100–1 × 10^8^); 9/12 ^b^
**NaCl**	1 × 10^8^ [1 × 10^8^–1 × 10^8^] (1 × 10^8^–1 × 10^8^); 12/12 ^b^	1 × 10^8^ [1 × 10^8^–1 × 10^8^] (1 × 10^8^–1 × 10^8^); 12/12 ^c^	1 × 10^8^ [1 × 10^8^–1 × 10^8^] (1 × 10^8^–1 × 10^8^); 12/12 ^b^
**i-PRF**	1 × 10^8^ [1 × 10^8^–1 × 10^8^] (1 × 10^8^–1 × 10^8^); 12/12 ^b^	1 × 10^8^ [1 × 10^8^–1 × 10^8^] (10,000–1 × 10^8^); 10/12 ^bc^	1 × 10^8^ [1 × 10^8^–1 × 10^8^] (1 × 10^8^–1 × 10^8^); 12/12 ^b^
**i-PRF + Amoxicillin**	1 × 10^8^ [1 × 10^8^–1 × 10^8^] (1 × 10^8^–1 × 10^8^); 12/12 ^b^	10 [1–100] (1–1 × 10^8^); 1/12 ^a^	1 × 10^8^ [1 × 10^8^–1 × 10^8^] (1 × 10^8^–1 × 10^8^); 12/12 ^b^
**Kruskal–Wallis H**	58.07	41.80	50.82
**df**	4	4	4
***p*-value**	<0.001	<0.001	<0.001
**Effect size, ε^2^**	0.983	0.687	0.851

Note: Superscript letters (a–c) indicate the results of Bonferroni-adjusted post hoc pairwise comparisons following the Kruskal–Wallis test. Values sharing the same superscript letter are not significantly different, whereas values with different superscript letters are significantly different.

**Table 4:** Inter-species comparison of residual CFU/mL values within each treatment group after the 24-h residual exposure/incubation period.

Treatment	*Streptococcus mutans* Median [IQR] (Min–Max)	*Staphylococcus aureus* Median [IQR] (Min–Max)	*Enterococcus faecalis* Median [IQR] (Min–Max)	H	df	*p*-Value	Epsilon^2^
**CHX**	550 [7.75–257,500] (1–1 × 10^6^) ^a^	1 [1–1] (1–1 × 10^8^) ^b^	5.5 [1–10] (1–100) ^ab^	9.21	2	0.010	0.218
**LLLT**	1 × 10^8^ [1 × 10^8^–1 × 10^8^] (1 × 10^8^–1 × 10^8^) ^a^	55,000 [775–1 × 10^8^] (10–1 × 10^8^) ^b^	1 × 10^8^ [7.50 × 10^7^–1 × 10^8^] (100–1 × 10^8^) ^ab^	10.09	2	0.006	0.245
**NaCl**	1 × 10^8^ [1 × 10^8^–1 × 10^8^] (1 × 10^8^–1 × 10^8^) ^a^	1 × 10^8^ [1 × 10^8^–1 × 10^8^] (1 × 10^8^–1 × 10^8^) ^a^	1 × 10^8^ [1 × 10^8^–1 × 10^8^] (1 × 10^8^–1 × 10^8^) ^a^	0.00	2	1.000	0.000
**i-PRF**	1 × 10^8^ [1 × 10^8^–1 × 10^8^] (1 × 10^8^–1 × 10^8^) ^a^	1 × 10^8^ [1 × 10^8^–1 × 10^8^] (10,000–1 × 10^8^) ^a^	1 × 10^8^ [1 × 10^8^–1 × 10^8^] (1 × 10^8^–1 × 10^8^) ^a^	4.11	2	0.128	0.064
**i-PRF + Amoxicillin**	1 × 10^8^ [1 × 10^8^–1 × 10^8^] (1 × 10^8^–1 × 10^8^) ^a^	10 [1–100] (1–1 × 10^8^) ^b^	1 × 10^8^ [1 × 10^8^–1 × 10^8^] (1 × 10^8^–1 × 10^8^) ^a^	29.67	2	<0.001	0.838

Note. CHX: chlorhexidine; LLLT: low-level laser therapy; i-PRF: injectable platelet-rich fibrin; CFU: colony-forming unit; IQR: interquartile range. Ceiling n/12 indicates the number of samples recorded at the predefined upper recording limit of 1 × 10^8^ CFU/mL. Within each bacterial species, treatment groups that do not share the same superscript letter are significantly different according to Bonferroni-adjusted Mann–Whitney U tests. Effect size was calculated as epsilon-squared.

## Data Availability

The original contributions presented in this study are included in the article. Further inquiries can be directed to the corresponding author.

## Abbreviations

The following abbreviations are used in this manuscript:

ASA	American Society of Anesthesiologists
ATCC	American Type Culture Collection
BHI	Brain Heart Infusion
CFU	Colony-forming unit
CHX	Chlorhexidine
CW	Continuous wave
i-PRF	Injectable platelet-rich fibrin
LLLT	Low-level laser therapy
NaCl	Sodium chloride
PRF	Platelet-rich fibrin
rpm	Revolutions per minute
SBA	Sheep blood agar
